# Modified Surface Relief Layer Created by Holographic Lithography: Application to Selective Sodium and Potassium Sensing

**DOI:** 10.3390/s19051026

**Published:** 2019-02-28

**Authors:** Sabad-E Gul, Luke O’Neill, John Cassidy, Izabela Naydenova

**Affiliations:** 1 Centre for Industrial and Engineering Optics/School of Physics and Clinical & Optometric Sciences, College of Sciences and Health, Technological University Dublin, Dublin D08 NF82, Ireland; sabade.gul@mydit.ie; 2 School of Chemical and Pharmaceutical Sciences, College of Sciences and Health, Technological University Dublin, Dublin D08 NF82, Ireland; john.cassidy@dit.ie; 3 FOCAS, Technological University Dublin, Dublin D08 NF82, Ireland; luke.oneill@dit.ie

**Keywords:** holographic sensors, diffractive optical sensors, biomedical sensors, sensors, holography, photopolymers, potassium ion detection, sodium ion detection, blood serum, PVC, sol gel

## Abstract

Point-of-care diagnostics will rely upon the development of low-cost, noncomplex, and easily integrated systems in order to examine biological samples such as blood and urine obtained from the patient. The development of metal ion sensors is a subject of significant relevance for physiological samples. The level of different blood electrolytes, mainly H^+^, Na^+^, K^+^ and Cl^−^ is considerably used to monitor irregular physiologies. The particular challenge in biosensing, and in fact for any other sensor, is signal differentiation between non-specifically bound material and the specific detecting of the target molecule/ion. The biosensors described in this paper are fabricated by a holographic recording of surface relief structures in a photopolymer material. The surface structures are modified by coating with either dibenzo-18-crown-6 (DC) or tetraethyl 4-tert-butylcalix[4]arene (TBC), which are embedded in a polymer matrix. Interrogation of these structures by light allows indirect measurement of the concentration of the analyte. The influence of polymer matrices with different porosities, plasticised polyvinyl chloride (PVC) and a sol-gel matrix, on the performance of the sensors for detection of K^+^ and Na^+^ is examined. Here we demonstrate a proof of concept that by using a matrix with higher porosity one can increase the sensitivity of the sensor. The results showed that the DC sensing layer provides a selective response to K^+^ over Na^+^ and the TBC modified grating is more responsive to Na^+^ over K^+^. The sensor responds to K^+^ and Na^+^ within the physiological concentration ranges.

## 1. Introduction

There is an urgent need for low-cost mass producible clinical diagnostic devices that allow measurements on site. Much effort has been devoted to decreasing the expense and time allocated to the measurement of physiologically important ions. The development of selective metal ions sensors is a focus of significant interest because of their clinical relevance. In future biosensors will become an essential part of modern healthcare because the demand for personalised medicine is increasing [1]. Moreover, point-of-care devices are cheaper diagnostic tools. In addition, many challenges of the healthcare system can be resolved by new sensor technologies. Therefore, it is expected that biosensors will become essential in the future [2]. Biosensor development is prompted by the development of new materials that can be used as functionalising materials [3].

The present research aim is to develop a holographic sensor platform, which will allow an increase in sensitivity and selectivity, lower operating costs, and facilitate the development of portable devices. In this article, we demonstrate the potential of our approach. The levels of blood electrolytes, particularly H^+^, K^+^, Na^+^ and Cl^−^, are widely used to monitor aberrant physiologies related with pulmonary emphysema, acute and chronic renal failure, heart failure and diabetes [4]. Particularly K^+^ ion analysis is challenging due to interference from high concentrations of Na^+^ in blood. It has been previously found that crown ethers have a remarkable capacity to form stable complexes [5] by certain cations, mainly with alkali and alkaline earth ions. In the present research, dibenzo-18-crown-6 (DC) was used as an ionophore to sense K^+^ ions. Previously, reflection holograms have been fabricated for biomedical sensing application in a silver halide holographic recording material. The incorporation of chelating agent 18-crown-6 was investigated to develop an ion-selective sensor. The sensor was shown to be able to determine K^+^ concentrations in physiological ranges and nearly unaffected by variations in Na^+^ [6,7]. Reflection holograms are inherently more difficult to mass produce than transmission holograms due to the requirement for extreme mechanical stability during manufacturing. In addition, the use of silver halide holographic recording material implies a higher cost of the sensor which may be prohibitive in everyday use. In order to overcome these challenges, we propose the use of transmission surface relief grating (SRG) fabricated in a low-cost self-developing photopolymer material, which production does not require stability on a nanometer scale.

The development of sensor technology platform based on surface relief gratings has been attracting significant attention recently [8,9,10]. Hsiao et al [8] developed a surface sensor platform for biosensing based on nanoporous polymeric gratings modified with aminopropyltriethoxysilane. Modification with aminopropyltriethoxysilane leads to immobilization of biomolecules onto the polymeric surface grating. The authors reported the detection of several biomolecules including biotin, streptavidin, biotinylated anti-rabbit IgG, and rabbit-IgG [8]. Ye et al. reported the fabrication of a novel sensing method based on diffraction gratings by using poly (acrylic acid) hydrogel modified with glucose oxidase. The sensor was developed for quantitative glucose detection [9]. Z. Zhou and co-workers demonstrated a proof-of-principle of sensing application based on bioactive diffractive optical elements (DOEs) fabricated by using functionalized silk fibroin films [9]. 

**Figure 1:**
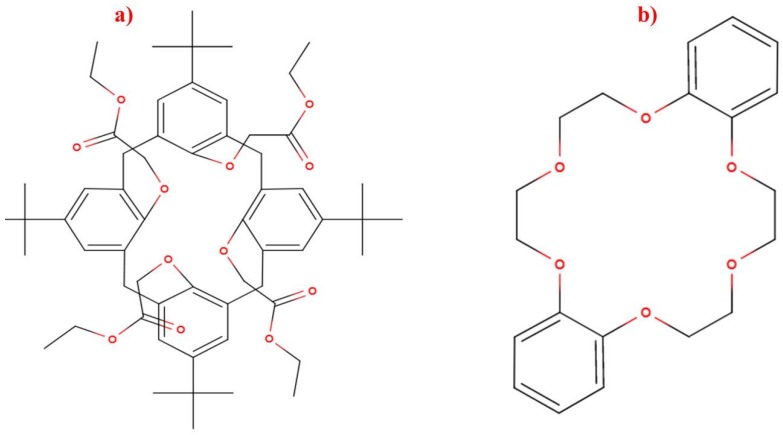
Structures of (a) tetraethyl 4-tert-butylcalix[4]arene (TBC) and (b) dibenzo-18-crown-6 (DC).

The sensor reported here is created by holographic lithography used to create SRG in a self-processing photopolymer material. This method is particularly useful when a flexible design of the sensing structures is required. A variety of spatial frequencies can be achieved by simply changing the angle between the two recording beams. More complex surface patterns can be achieved by using more than two beams or by multiplexing more than one optical pattern in the same location of the material. The surface relief amplitudes can be tuned by varying the exposure time or photopolymer chemical composition. It has been previously demonstrated that the initial surface relief modulation is very important for the performance of the sensor as it determines the starting diffraction efficiency and the dynamic range of the sensor [11]. The gratings reported in this paper were modified by incorporation of either DC or TBC (Figure 1) as a chelating agent in plasticised polyvinyl chloride binder or in a sol-gel matrix for the detection of potassium and sodium. To the best of our knowledge, the proposed sensing structures have been demonstrated and studied for the first time. Interrogation of these structures by light allows indirect measurements of chemical analytes’ concentration in real time. Some of the particular advantages of the proposed sensor are that the diffraction efficiency is not dependent on the polarisation of the probe beam, which is a significant issue in surface plasmon resonance sensors. One can argue that in order to achieve high accuracy the intensity of the probe beam needs to be highly stable. Laser sources providing such high stability are expensive and not suitable for low-cost sensing systems. This issue can be resolved by measuring the intensity of both beams, probe beam and diffracted beam simultaneously, by the incorporation of a low-cost beam splitter. The two ionophores used in this article have been widely used in the fabrication of ion-selective electrodes for potentiometric measurements [12,13,14]. Ion-selective potentiometry is a popular electro-analytical method [15,16] and there are a number of applications which include, but are not limited to the detection of ions in water or other media. Ion-selective electrodes are a norm in the industrial sector, for the physiological measurements and environmental monitoring. However, such potentiometry comes at a cost of potential deviations from the Nernst theory as it depends on the variation of temperatures in the surrounding environment, and potential drift and compensation of ion activity. Ionic strength adjustment buffers can be used to accommodate activity and flow through systems can alleviate potential drift. Furthermore, the spectrophotometric approach can become complicated when conflicting ions are present and it may require a considerable increase in time with reference to applications of ion-selective potentiometry. Therefore, it is important to develop a sensor which is less complex, not strongly influenced by the presence of other ions and easily used by non-specialists [17]. 

Sol-gel technology is an existing innovation in science that needs a multidisciplinary approach for its numerous applications. Sol gel is the process of making crystalline and amorphous materials at low temperature. The doping of various analyte sensitive materials during the formation of more ordered matrices with controlled porosity can be possible in this process [18]. The key advantages of sol-gel layers are that they have a high surface area which helps in sensing applications. While the development of Na^+^ and K^+^ sensors is the aim of this work, this will demonstrate the feasibility of the modified SRG for broader applications.

## 2. Materials and Methods

The synthesis procedure of acrylamide based photopolymer has been previously presented [19,20]. All the chemicals used in this work were of analytical grade, they were purchased from Sigma Aldrich, and used without further modification. The composition of the photopolymers that were used during this experiment is presented in Table 1 below.

**Figure 2:**
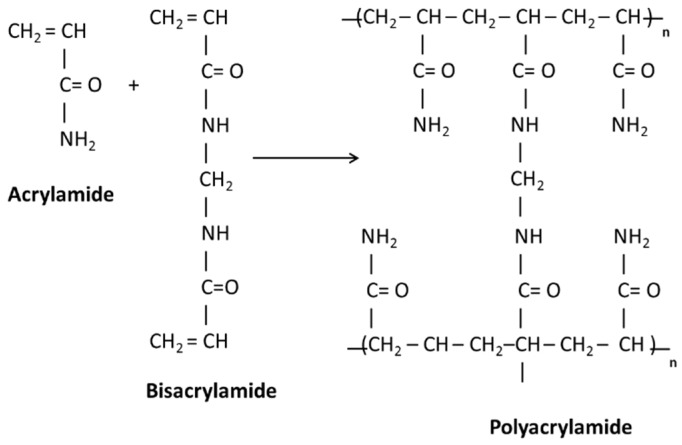
Crosslinking reaction between acrylamide and bisacrylamide to produce crosslinked polyamide.

The function of bisacrylamide crosslinker in the photopolymer composition is to bind the polymer chains together during the photoinduced photopolymerization reaction. This restricts the diffusion of the polymer chains out of the illuminated regions, thus maximising the refractive index modulation of the recorded grating. The mechanism for the crosslinking of acrylamide polymer chains by bisacrylamide is shown in Figure 2.

### 2.1. Fabrication of Surface Relief Gratings (SRG)

**Figure 3:**
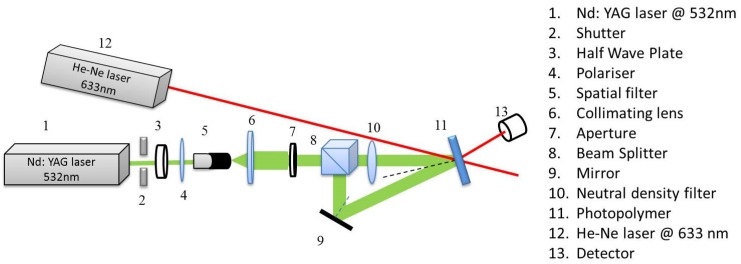
Experimental set-up for recording of the SRG.

The set up used for recording the holographic gratings as shown in Figure 3 was a double beam holographic lithography set up. The holograms were recorded at a spatial frequency of 300 lines/mm ±10. The total recording intensity in the two beams was 10 mW/cm^2^ and the recording time was 100 s. The recording light source was Nd: YVO4 laser emitting at 532 nm. (Coherent, Model Verdi 5W). Details of the optimisation of recording conditions were reported previously [21]. Helium-neon laser (Uniphase, Model No 106-1) emitting at wavelength 633 nm was used in order to obtain the Bragg selectivity curves of the recorded gratings. The intensity of the diffracted beam was measured by using an optical power meter (Newport 1830-C). The grating was positioned on a rotational stage in order to measure its diffraction efficiency variation with the variation of the incident angle of the probe beam. The accuracy of the rotation stage was 1 × 10^−3^ deg. An optical power meter was used in order to measure the first order diffracted beam intensity of the recorded gratings. The diffraction efficiency immediately after recording was purely determined by the volume diffraction grating and the surface relief amplitude before exposure to elevated temperature was measured to be <1 nm. The diffraction efficiency after exposure to elevated temperature is mainly determined by the surface relief structure. The diffraction efficiency *η* was recorded at Bragg angle, and defined as the ratio of the intensity in the first diffraction order *I_d_* and the incident intensity *I_in_* of the probe beam:

(1) $$\eta =\frac{I_{d}}{I_{in}}\times 100$$

The proposed sensor utilises SRG. It has been previously observed [21] that the relationship of the period, *Λ*, and surface relief amplitude, *d*, of the SRG are controlled by the recording conditions. Based on these parameters and the wavelength at which it operates, the regime of operation of the SRG can be determined with the help of the Klein-Cook *Q* parameter [22] as defined below:

(2) $$Q=\frac{2\pi \lambda_{r}d}{n\Lambda^{2}}$$

Whereby, n represents the refractive index of the recording medium and $\lambda_{r}$ represents the reconstruction wavelength. The values of Klein-Cook *Q* above 10 relate to thick gratings and are described by using Kogelnik’s coupled wave theory [23]. However, gratings characterised by *Q* < 1 are considered as being thin and described using Raman–Nath theory [24]. The results here for SRG generate *Q* < 1 thus the sensor operates as a thin grating.

The diffraction efficiency of the structure was measured simultaneously with the surface relief amplitude at each stage of preparation of the sensor and after its exposure to the analyte. This information was used to estimate the contribution of both the surface and the volume component of the photonic structure on the *η* of the sensor device. The *η* after exposure to a higher temperature is mainly determined by the surface relief structure. The η of the surface relief grating was calculated using Equation (3) [23]:

(3) $$\eta =J_{m}^{2}\left(\frac{\pi \Delta nd}{\lambda_{r}}\right)$$

where $J_{m}$ is the Bessel function of the order *m*. The *I_in_* beam is diffracted into a number of orders and the diffracted amplitude in the *m*^th^ order is given by the relevant Bessel function. Whereas Δn is the refractive index modulation of the grating, which is determined by the difference in refractive index of the peaks and the troughs of the SRG.

For the surface relief structure studied here ∆*n* = 0.5 (refractive index of the photopolymer material is 1.5 and refractive index of air is 1) and *d* = 400 nm. For a probe beam of wavelength 633 nm, the diffraction efficiency of purely structure was estimated to be 33%. The measured diffraction efficiency after thermal exposure was 35%. This implies that the diffraction efficiency after thermal treatment is mainly determined by the surface relief structure. This could be also partially explained by the fact that the thickness of the layers has decreased from 60 μm to 22 μm after exposure to higher temperature. The process of the grating formation was reported previously [21,25]. The detailed process of SRG fabrication published elsewhere [19,20]. The modification of the layer in this study is targeting selective detection of Na^+^ and K^+^ and is described in Section 2.5.

### 2.2. AFM

All samples were examined at ambient conditions by using an atomic force microscope (AFM) a MFP-3D BIO. The cantilevers used in this experiment were 240 μm long, the resonant frequency was 70 kHz and the force was kept constant at 2 N/m. During the examination of the gratings, AFM was operated in alternate contact AC mode in order to minimize tip-sample interaction. 

### 2.3. SEM

Scanning electron microscopy (SEM) analysis was performed on all test materials in order to analyse surface topography and porosity. Hitachi SU 6600 Fe-SEM instrument (Hitachi high Technologies Europe GMBH Whitebrook Park Lower Cookham Road Maidenhead SL6 8YA UK) was used in this study. Calibration of SEM was performed with Au on carbon standard provided by Agar Scientific (Essex, UK). Samples were prepared via spin coating onto glass slides at 1000 rpm and imaged at an accelerating voltage of 5 kV and a working distance of 10 mm. 

### 2.4. Preparation of Sensing Layer

**Figure 4:**
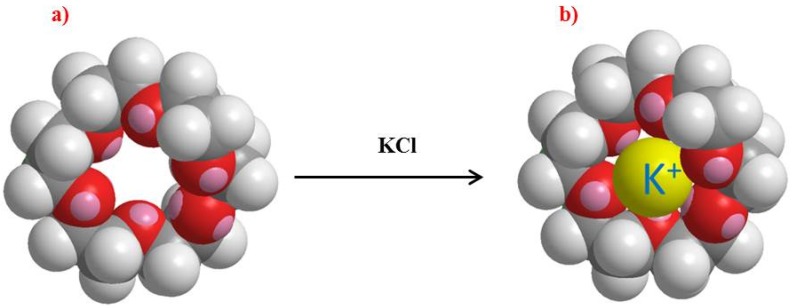
(**a**) 18-crown-6 ether (**b**) 18-crown-6 ether after exposure to KCl solution.

The target ions to be detected are potassium and sodium. Potassium and sodium ions are most commonly analysed by clinical laboratories. The size of the 18-crown-6 is the right fit for potassium ions and this provides selectivity to them, shown in schematic in Figure 4 [12]. 

A relatively new class of ionophores, calixarenes, was introduced after the 1990s [26]. Na^+^ selective membranes with incorporated Calixarene ionophores (TBC) are well-known as best sodium ionophores. 

#### 2.4.1. DC or TBC in PVC Matrix

The surface holograms were modified with a sensing layer with Calixarene ionophores (TBC) immobilised in a PVC plasticised matrix [27,28] or dibenzo-18-crown-6 (DC) [29,30]. The composition of chemicals that were studied during this experiment is presented in Table 2 below.

All the components were stirred for 2 h in 20 mL of tetrahydrofuran (THF) at 60 °C until all the chemicals were completely dissolved in the mixture. Then this solution was spin coated on the SRG at 1000 rpm and the solvent allowed to evaporate to form a layer. Next, the gratings were exposed to the K^+^ and Na^+^ in water solutions to evaluate their response after different time intervals. 

#### 2.4.2. DC or TBC in a Sol-Gel Matrix

The SRG was coated with tetraethyl orthosilicates (TEOS, 98%) containing DC or TBC. The sol-gel method from TEOS provides mesoporous matrices that allow chemical species (in this study cations such as K^+^ and Na ^+^) to diffuse easily and to interact with analyte sensitive material and thus provide for faster response of the sensor. The synthesis of the layers by the sol-gel procedure has been previously reported [19,31]. The amount of DC and TBC varied from 0.05 g to 0.1 g. The optimisation involved finding the maximum amount of DC and TBC that can be incorporated in a layer while retaining good optical quality. The solution was spin-coated at 500 rpm on the pre-recorded surface grating. Next, the coated samples were left for 24 h at room temperature before further studies. Then the gratings were exposed to solutions with different molar concentrations of K^+^ and Na^+^ for pre-determined time intervals. The gratings were studied under AFM and the change in diffraction efficiency measured at different stages.

## 3. Results and Discussions

### 3.1. Characterisation of Holographic Surface Relief Structures and Sensing Layer Material by AFM

**Figure 5:**
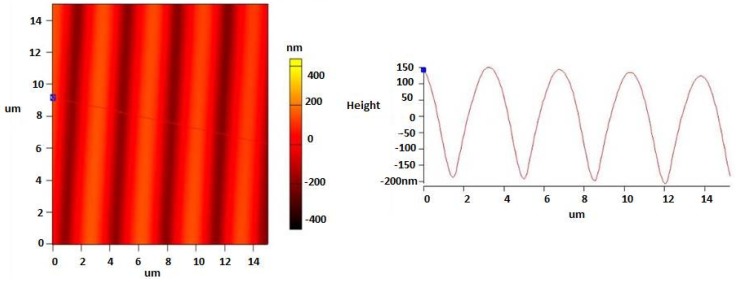
Two-dimensional AFM scans of the SRG after thermal treatment.

AFM was used for characterizing the surface topography. The surface relief profiles are presented in Figure 5. The amplitude observed in the structures of SRG is in the range of 350–400 nm, and line spacing 3.54 µm ± 3% was observed by AFM. 

**Figure 6:**
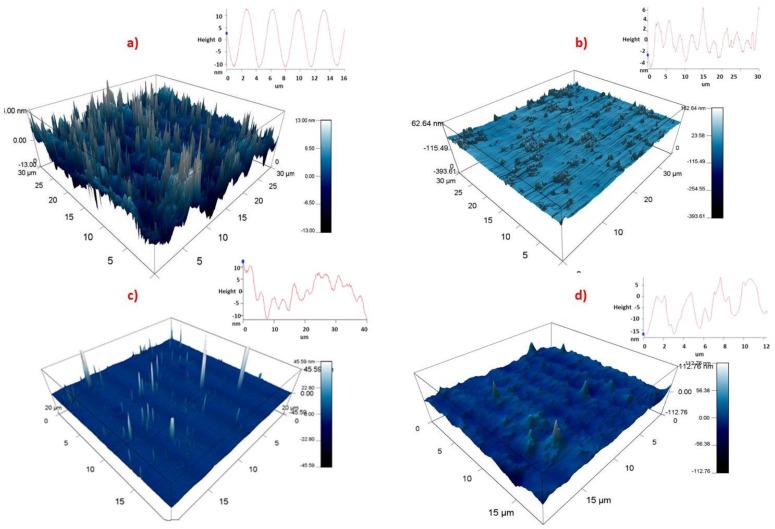
(**a**) Three-dimensional AFM scans of the SRG coated with DC in PVC; (**b**) coated with TBC in PVC; (**c**) coated with DC in a sol-gel matrix; (**d**) coated with TBC in a sol-gel matrix.

The gratings were characterized before and after coating with the sensing layer. The sensing layer contains a chelating agent DC or TBC in PVC. After the surface structure was coated with DC the amplitude decreased from 350–400 nm to 20 nm approximately (Table 3) which clearly indicated that the troughs were filled with the sensing material. A separate set of samples were coated with TBC. These samples were studied by AFM and it was observed that their SRG amplitude decreased from 350–400 nm to 10 nm approximately as can be seen in Figure 6.

### 3.2. Diffraction Efficiency and Angular Selectivity Studies

**Figure 7:**
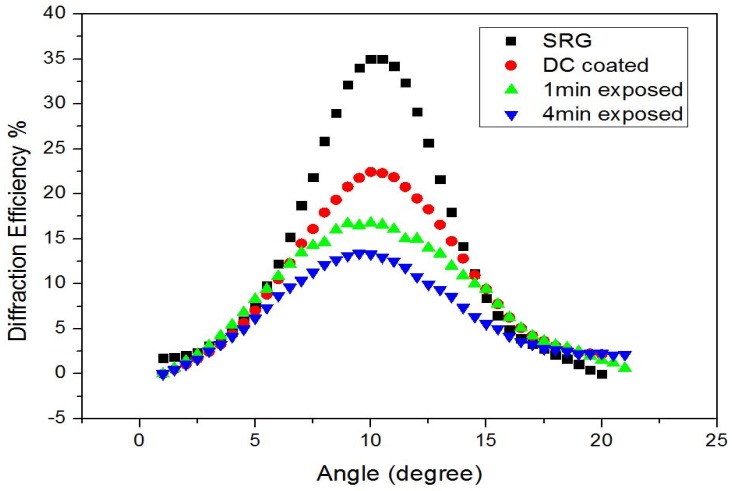
Bragg curves for SRG (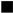), after spin coating with (DC)/PVC layer (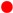) and after exposure to analyte K^+^ (50 mM) (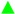,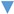).

The maximum diffraction efficiency of 60% was achieved for a photopolymer grating after holographic recording as listed in Table 3. The high value of diffraction efficiency is a result of the volume diffraction grating as the SRG has negligible amplitude before thermal treatment. After thermal treatment, the diffraction efficiency was reduced to ~35% and a shift of the Bragg angle was observed. The shift is observed in the photopolymer layer after thermal treatment possibly due to an increase in the effective refractive index as the density of the layer is clearly increased after thermal treatment (the thickness of the layer was measured to decrease from 60 to 22 μm). Other possible causes are shrinkage of the layer after thermal treatment causing a variation of the spatial frequency of the surface structures. The Bragg angle selectivity curve was measured for layers modified with analyte sensitive materials. Diffraction efficiency further decreased after coating due to the substitution of the air in the troughs with material with a higher refractive index. Figure 7 shows example sets of experimental curves for the diffraction efficiency as a function of incident angle for a holographic grating at different stages of the experiment, i.e., before thermal treatment, after thermal treatment, after coating with the DC sensing material, and after exposure to the analyte. The change in the peak value of the diffraction efficiency was followed as an indicator of the presence of the analyte. 

In order to evaluate the response of the structure to the analyte, measurements of the peak diffraction efficiency were taken at different times.

### 3.3. Evaluation of the Selectivity and Sensitivity Response of the Sensor Achieved by Coating with DC or TBC in Polyvinyl Chloride (PVC) Matrix

#### 3.3.1. Evaluation of the Selectivity in PVC Matrix

**Figure 8:**
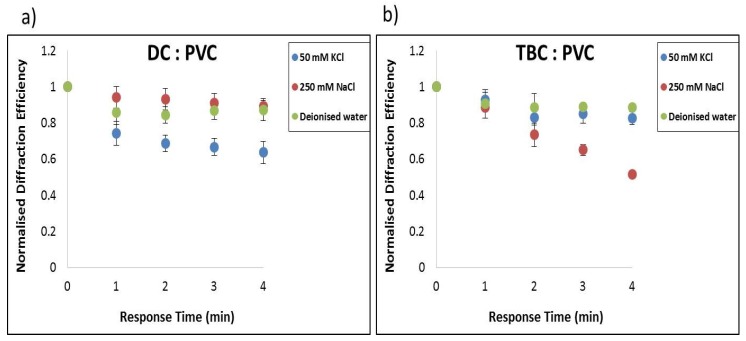
Comparison of relative response in terms of normalised DE change of (**a**) DC to 250 mM NaCl, 50 mM KCl and deionised water and (**b**) TBC coated structures to 50 mM KCl, 250 mM NaNO_3_, and deionised water.

In order to determine the selectivity of the proposed holographic biosensor for detection of the target analytes K^+^ and Na^+^, solutions with different concentrations (C) of KCl and NaNO_3_ were prepared. Their association to become a close fit for the cation to the crown cavity and the potential to form stable complexes is a known property of crown ethers. Inspection of Figure 8a reveals that the dibenzo-18-crown-6 gives a dominant response for K^+^ ion concentration, while the sensor showed a minute response to Na^+^ and the reference water sample [7]. The diffraction efficiency drops for K^+^ from 1 to 0.6, whereas for Na^+^ it drops down only from 1 to 0.9 although the Na^+^ concentration was five times higher than the K^+^ concentration.

The results presented in Figure 8b show that the TBC on exposure to Na^+^ ions yield a dominant response over K^+^ ion concentration, irrespective of the anion, while the hologram showed little response to deionised water (DI) and K^+^. It was observed that the nature of the anion (NaCl or NaNO_3)_ does not have a significant effect on the response of the sensor. The diffraction efficiency drops for Na^+^ from 1 to 0.52, whereas for K^+^ it drops down from 1 to 0.8 only. The Na^+^ concentration is five times higher than K^+^ because Na^+^ physiological ranges are higher than K^+^. The aim of these studies is to detect physiological ranges of blood serum which is 3.5–5.0 mM for K^+^ [32]. The results indicated that with the PVC matrix the lowest detected molar concentration is 10 mM K^+^ and needs further improvement, which is addressed in Section 3.4.

#### 3.3.2. Evaluation of the Sensitivity in PVC Matrix Containing DC

**Figure 9:**
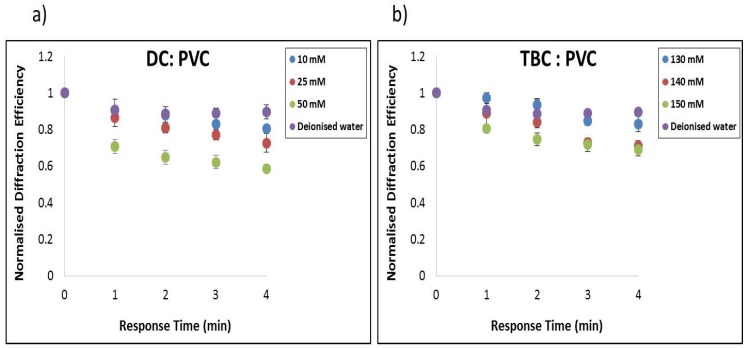
Normalised diffraction efficiency change response DC and TBC plasticised PVC photonic structures (**a**) KCl exposure to (10–50 mM) (**b**) NaNO_3_ exposure to physiological levels (130–150 mM).

The aim of this study is to detect physiological ranges of potassium and sodium in serum [4]. Different molar concentrations solutions were prepared for KCl. The results in Figure 9a are from testing the layers when exposed to the prepared molar solutions 10–50 mM of K^+^ and deionised water (DI). As can be seen the normalised diffraction efficiency changes as the exposure time increases and molar concentration increases (Figure 9a). The largest change is for 50 mM K^+^ as the normalised diffraction efficiency drops down from 1 to 0.6. In water it drops down only from 1 to 0.9.

The normal levels of Na^+^ serum in humans are in the range of 135–148 mM [32]. Solutions of NaNO_3_ were prepared with three different molar concentrations 130, 140 and 150 mM. The sensor based on the TBC ionophore was exposed to concentrations 130–150 mM and deionised water. As can be seen in Figure 9b the normalised diffraction efficiency change as the exposure time and molar concentration of the solution increases. There is a dominant response for higher molar concentration which is 150 mM as the normalised diffraction efficiency drops down from 1 to 0.7. In deionised water it drops down from 1 to 0.9, whereas in lowest detected molar concentration (130 mM) the normalised diffraction efficiency drops down from 1 to 0.86. The result implies that there is a need to improve the sensitivity toward Na^+^ in order to distinguish better the signal at 130 mM concentration from the background change caused by deionised water.

### 3.4. Improvement of the Sensitivity by Changing the Porosity of the Matrix

#### 3.4.1. SEM Characterisation

**Figure 10:**
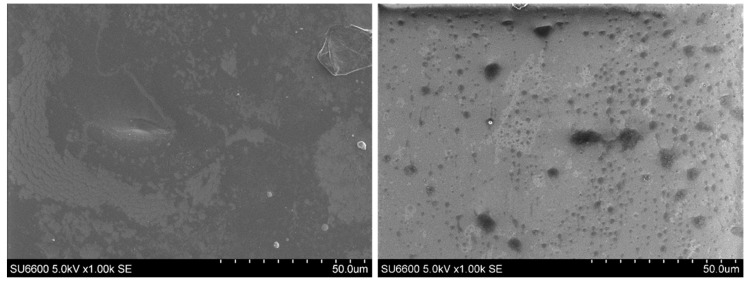
Demonstration of the porosity of the matrix by comparing two matrices (**a**) SEM image of PVC + DOPT; (**b**) Sol-gel.

The aim here was to change the PVC matrix with a matrix with increased porosity for achieving physiological ranges of potassium, which was not possible in PVC membrane, and improve the differentiation between background signal and response of the sensor to 130 mM Na^+^. SEM images were obtained of the thin films coated on a glass surface of PVC + DOPT and Sol-gel (Figure 10) in order to compare the porosity of the two materials. It is clearly illustrated in the SEM images that a sol-gel matrix has a porous structure. The porous structure of the sol gel will allow for the analytes to easily penetrate into the matrix.

#### 3.4.2. Evaluation of the Selective Response of the Sensor Achieved by Coating with DC in a Sol-Gel Matrix

**Figure 11:**
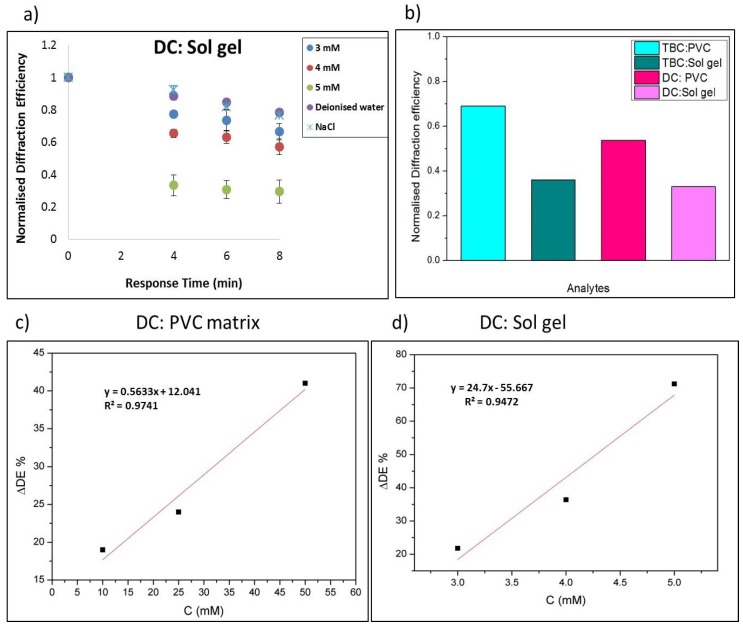
(**a**) Normalised diffraction efficiency change response of DC coated photonic structures in a sol-gel matrix exposure to physiological levels (3–5 mM) KCl, DI and 130 mM NaCl, Sensitivity of the response at 4 min for DC, (**b**) comparison of change in normalized diffraction efficiency in PVC and sol-gel matrices at 4 min data points, (**c**) sensitivity of the structure determined at 4 min for DC in plasticised PVC (10–50 mM) and (**d**) sensitivity of the structure determined at 4 min for DC in a sol-gel matrix (3–5 mM).

Similarly, solutions of KCl were prepared in order to determine the selectivity of the sensor with the sol-gel layer. Figure 11a shows that the dibenzo-18-crown-6 on exposure to K^+^ ions give a dominant response over the Na^+^ ion concentration, while the sensor showed a minute response to DI. Three different molar concentrations of KCl were prepared and exposed to the sensors along with the PVC membrane coated sample. The results in Figure 11a are from testing the structures when exposed to 3–5 mM KCl and deionised water. The normalised diffraction efficiency changes as the exposure time increases (Figure 11a). There is a dominant response for 5 mM K^+^ as the normalised diffraction efficiency drops down from 1 to 0.244. In DI it drops down only from1 to 0.78 and from 1 to 0.76 for NaCl. The sensor was coated with DC-PVC or DC-sol gel and the sensitivity of the sensor was evaluated. The gratings were exposed to the analyte for different time intervals. The sensitivity for PVC matrix at 4 min exposure to the analyte (DE/ mM) is 0.5 %/mM which were observed for 10–50 mM concentrations Figure 11c. The sensitivity at 4 min exposure to the K^+^ analyte (DE/mM) is 24.7 %/mM, which was observed for 3–5 mM in a sol-gel matrix modified structure, Figure 11d. Comparison of the change in diffraction efficiency for structures coated with DC in the two matrices can be seen in Figure 11b. 

#### 3.4.3. Evaluation of the Selective Response of the Sensor Achieved by Coating with TBC in a Sol-Gel Matrix

**Figure 12:**
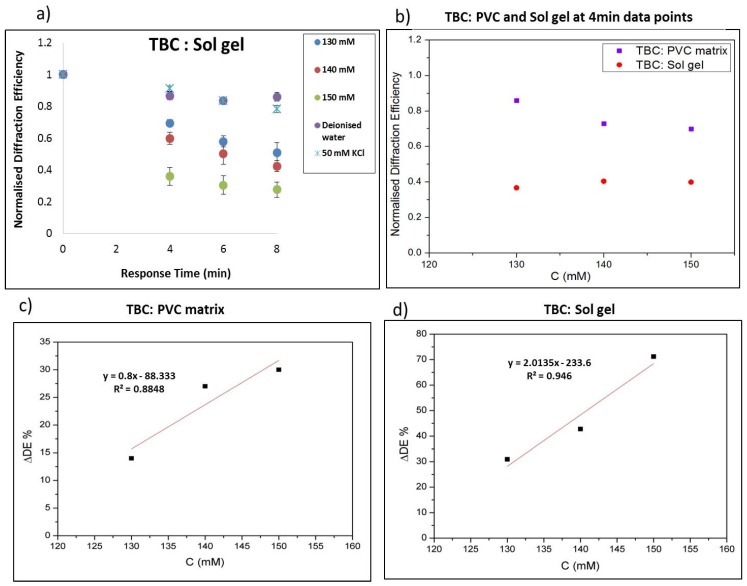
(**a**) Normalised diffraction efficiency change response TBC photonic structures in sol-gel layers exposure to (130–150 mM) Na^+^, (**b**) comparison PVC and Sol gel at 4 min data points, (**c**) Sensitivity of the response at 4 min for TBC in PVC and (**d**) sol gel.

Next, the samples were modified with TBC in a sol-gel matrix. The sensor based on the TBC ionophore was exposed to concentrations of 130–150 mM NaNO_3_. In Figure 12a it can be seen clearly that the normalised diffraction efficiency changes as the exposure time increases. In Figure 12b data show the response at 4 min of exposure to the analyte for both layers –TBC in PVC matrix and TBC in a sol-gel matrix. There is a dominant response of the sol-gel layer as compared to the PVC layer. Comparison of the change in diffraction efficiency for structures coated with TBC in the two matrices can also be seen in Figure 11b. Next, the sensitivity at 4 min exposure to the analyte (DE/mM) was estimated to be 0.8 %/mM which was observed for 130–150 mM physiological concentrations Figure 12c for the PVC matrix. The sensitivity at 4 min exposure to the Na^+^ analyte (DE/mM) is 2.0 % / mM which were observed for 130–150 mM in a sol-gel matrix, Figure 12d. In Figure 11 and Figure 12 there are only three points on the calibration curve; the aim of this work is to demonstrate the feasibility of the sensor, rather than rigorously validate it as an analytical technique.

## 4. Conclusions

Holographic sensors were fabricated for the detection of physiologically relevant metal ions. SRGs were modified with DC or TBC. DC shows a selective response of the devices to K^+^ over Na^+^, whereas for TBC there is a dominant response for Na^+^ over K^+^. The sensors respond to Potassium or Sodium metal ions within the physiological ranges. Normal levels of Na^+^ in human serum lie in the range 135–148 mM and the normal K^+^ level is 3.5–5.0 mM [33]. It was demonstrated that the sensitivity of the structures can be significantly increased by substituting the PVC matrix with a sol-gel matrix with larger porosity. While the results demonstrate that the modified SRG can sense K^+^ and Na^+^, alternative sensing layers can also be used. These layers could comprise the array of ionophores, traditionally used in potentiometry or molecularly imprinted polymers. The advantage of such surface relief photonic structures is their ability to be modified for different analytes of interest by changing the modifying top layer. This novel transduction photonic structure provides a quick response, it is low-cost and can be integrated with portable sensing devices. 

## Figures and Tables

**Table 1:** Composition of the photopolymer.

Components	Chemical Structures	Amount (g)	% w/w in Dry Layer
Acrylamide	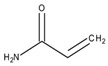	1.0	24.5
N,N′Methylenbisacrylamide	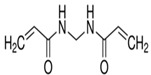	0.2	4.9
Polyvinyl alcohol (10% wt/wt)	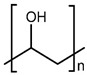	1.75	43.0
Triethanolamine	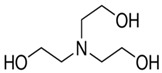	1.12	27.5
Erthrosine B dye (0.01% wt/wt)	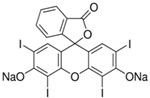	0.0044	0.1

**Table 2:** Composition of the coating layer.

Components	Amounts (g)	% w/w in Dry TBC Layer	% w/w in Dry DC Layer
Polyvinyl Chloride (PVC)	0.3	28.6	28.6
Dioctyl terephthalate plasticizer (DOTP)	0.6	57.1	57.1
Dibenzo-18-crown-6 (DC)	0.1	-	9.5
tetraethyl 4-tert-butylcalix[4]arene Ionophore (TBC)	0.1	9.5	-
Sodium tetraphenylboron	0.05	4.8	4.8

**Table 3:** Diffraction efficiency (DE %) and surface modulation of SRG at different stages of the experiment.

Diffraction Efficiency (DE %)	Surface Modulation (nm)
Photopolymer 60% ± 5%	<1
After thermal treatment 35% ± 3%	400
After coating with dibenzo-18-crown-6 (DC) 20% ±3% in PVC matrix	20
After coating with Tetraethyl p-tert-butylcalix[4]arene Ionophore (TBC) in PVC matrix 20% ± 3%	10
After coating with dibenzo-18-crown-6 (DC) in sol gel matrix 20% ± 3%	20
After coating with Tetraethyl p-tert-butylcalix[4]arene Ionophore (TBC) in a sol-gel matrix 20% ± 3%	18
